# Synthesis and characterization of Hyaluronic Acid (HA) modified polymeric composite for effective treatment of wound healing by transdermal drug delivery system (TDDS)

**DOI:** 10.1038/s41598-023-40593-9

**Published:** 2023-08-17

**Authors:** Hina Raza, Asmara Ashraf, Rahat Shamim, Suryyia Manzoor, Younas Sohail, Muhammad Imran Khan, Nadeem Raza, Nasir Shakeel, Komal Aziz Gill, Adel El-Marghany, Sikandar Aftab

**Affiliations:** 1 Faculty of Pharmacy, Bahauddin Zakariya University, Multan, Pakistan; 2 Punjab University College of Pharmacy Punjab University, Lahore, Pakistan; 3 Institute of Chemical Sciences, Bahauddin Zakariya University, Multan, Pakistan; 4 Department of Botany, Emerson University, Multan, Pakistan; 5 Research Institute of Sciences and Engineering (RISE), University of Sharjah, 27272 Sharjah, United Arab Emirates; 6 Department of Chemistry, Faculty of Science, Imam Mohammad Ibn Saud Islamic University (IMSIU), Riyadh, Saudi Arabia; 7 Faculty of Chemistry, Department of Materials Technology and Chemistry, University of Łódź, Łódź, Poland; 8 Division of Geochronology and Environmental Isotopes, Silesian University of Technology, 44-100 Gliwice, Poland; 9 Department of Chemistry, College of Science, King Saud University, P.O. Box 2455, 11451 Riyadh, Saudi Arabia; 10 Department of Intelligent Mechatronics Engineering, Sejong University, 209 Neungdong-ro, Gwangjin-gu, Seoul, 05006 South Korea

**Keywords:** Biochemistry, Chemistry

## Abstract

The present study aimed to fabricate a novel polymeric spongy composite to enhance skin regeneration composed of Nystatin (antifungal agent) and Silver Nanoparticles (AgNps). Different formulations (F1–F8) were developed & characterized by using various analytical techniques. AgNps synthesized by chemical reduction method showed spherical morphology 2 µm in size showed by SEM and XRD. A fine porous structure of gel embedded with AgNps having an amorphous structure with 10 % crystallinity due to AgNps was found. IR spectra revealed no chemical interaction between polymers and Nystatin. An increase in thermal stability of formulation was observed till 700 ℃ analyzed by Differential Scanning Calorimetry. Cytotoxic analysis on L929 mouse skin fibroblast cells showed a decrease in cell viability as Ag concentration increased (inactivating Fibroblast and keratinocytes) while 10 mg composition was found safest concentration (94%). Optimized formulation (F2) presented in-vitro drug release up to 90.59% ± 0.76 at pH 7.4, swelling studies (87.5% ± 0.57), water retention (26.60 ± 0.34), pH (5.31 ± 0.03). In the animal burn model, the group that received CHG/Ag/Nystatin healed the wound significantly (p < 0.05). These results suggested that optimized carrier can be used for other anti-fungal drugs facilitating the early healing of the wound.

## Introduction

Wound healing is a critical regeneration mechanism of ruptured tissue after getting an injury. Challenges and obstacles in the treatment of burnt skin using conventional methods demanding novel topical formulation with enhanced epithelial regeneration. The recent study aimed to encounter the creeping burn wounds by minimizing medication wave off from the burnt area leading to decreased drug contact time resulting in delayed regeneration^1^.To regenerate the tissues, medication essentially has good Spreadability, swelling, water absorption, and retention properties. Formerly different researchers focused on various strategies for enhanced wound healing using different polymers having bactericidal & fungicidal properties like PVA, PEG, gelatin, and collagen to boost the drug effect^2^. Hyaluronic Acid and Chitosan exhibit accelerated wound healing due to their unique nature. Chitosan encourages wound healing because of its hemostatic characteristics as it initiates the proliferation of fibroblasts, enhancing Infiltration & granulation, colonization of polymorph nuclear neutrophils and macrophages^1^. The properties of Hyaluronic Acid like Re-epithelization, soft tissue formation, good elasticity, and improved microvascular density make it suitable for wound healing. It also helps in the proliferation and migration of growing tissues which also improves wound healing^3^.

Recent research was performed to develop three polymeric spongy composites having good water absorption, retention, swelling properties, and Spreadability in addition to fungicidal and bactericidal effects. Drugs Nystatin and AgNps were introduced to increase the therapeutic efficacy of the composite. Ag+ has a bactericidal effect and Nano sizing enhances the ability to penetrate more easily into bacterial cell. Nystatin resides in BCS class IV having less permeability and less solubility, polymeric composite enhances the contact time of nystatin and releases the drug within 12 hours.

Burn wounds are the major devastating health crisis globally. According to WHO, Pakistan has a mortality rate of 55.9% due to a lack of rehabilitation and a warm climate that induces susceptibility to infections. Despite recent advances in anti-microbial therapy and wound management, infection and delayed healing is the most common problem. The use of topical antimicrobial agents decreases the mortality rate, but many agents used in the past are not effective now. So, to enhance the healing effect and decrease the chances of infections, a formulation was developed from Chitosan and Hyaluronic Acid both are cheap and bio-degradable having wound healing and anti-microbial properties, Nystatin and AgNps added for synergistic effect. To The best of our Knowledge, previously, no such combination was developed.

## Experiment methodology

### Chemicals

Chitosan (Molecular weight 500kDa, Degree of De-acetylation 85%), Silver nitrate (molecular weight 169.87 g/mole, purity ≥ 99.8%) Sigma Aldrich, Germany. l-Glutamic Acid (Molecular wt. 147.13g/mole) by Fluka Chemicals Switzerland. Hyaluronic Acid (analytical grade) SAFRiN laboratories Lahore, Pakistan. Ammonia (mol. Wt. 17.03 g/mole) BDH laboratory, England. Poly Vinyl Alcohol 1500 from Duksan pure chemicals Korea, Glucose (molecular weight 180.16g/mole) by Merck KGaA Germany. Acetic Acid (99–100% pure, mol. weight 60.05 g/mole) Merck Private Ltd. Karachi, Pakistan. Powder Nystatin purity 99% from ICI Pakistan LTD Pharmaceuticals Karachi, Pakistan. Male rats (white Albino) were arranged from Animal house (Department of Pharmacology) B.Z.U Multan. Micro-organisms: Candida Albicans, Staphylococcus Aureus.

Mouse skin fibroblast (NIH/3T3mouse fibroblast) was obtained from Panjwani Center for molecular medicine and Drug Research. Karachi, Pakistan.

### Development of CHG/Ag/nystatin composite

#### Synthesis of AgNps

AgNps were developed by using the chemical reduction method by Gusliani et al. Briefly Ag ions were developed by adding ammonium hydroxide and glucose in silver nitrate solution then by adding PVA at 60 ℃ and heating the solution until color changes (1B).

#### Fabrication of spongy composite

In recent study spongy composite (CHG/Ag/Nystatin) was fabricated by the crosslinking method. Briefly, Chitosan (0.5–1.5%) w/v was solubilized in 50ml acetic acid (0.5%) solution at 70 ℃ for 1hr (Solution A). Prepare 4% w/v 50ml clear solution of l-Glutamic Acid in 0.5% acetic acid by stirring at 70 ℃ (Solution B). Add solution B dropwise to solution A and stir it for 6 hr. until a viscous and clear solution was formed. Hyaluronic Acid (10 ml (0.2–0.5 % w/v) solution was added by continuous stirring. After that Nystatin, 0.1% w/v AgNps, and 0.4 % w/v Alpha tocopherol were added at room temperature and stir the mixture for 30–40 mins. The mixture was poured into a cuvette, kept at – 50 ℃ for 12 hours, and lyophilized for 24 hours. Add an equal weight of spongy composite in simple ointment B.P. to prepare ointment.

### Characterization

#### Organoleptic characteristics

CHG/Ag blank composite and CHG/Ag/Nystatin were visually inspected for color and odor. pH was determined by potentiometric method at a temperature of 25 ± 5 ℃. Viscosity was determined through Brookfield Viscometer using different spindles at room temperature.

#### Evaluation of porosity, water absorption, retention, and percent drug loading

The porosity of the spongy composite was assessed by the displacement method. Weighed dried sample (W_d_) was immersed in 50 ml ethanol solution for 24 hours. Weigh the sample (W_w_) again and calculate the porosity by using Eq. (1).

1 $$\mathrm{Porosity\,\, }(\mathrm{\%}) = \frac{{\mathrm{w}}_{\mathrm{w}}-{\mathrm{w}}_{\mathrm{d}}}{\mathrm{v}}\times 100.$$

Water absorption of composite was evaluated by Gravimetric method. Lyophilized weighed sample (W_d_) was dipped in water for 8 hours, draw the sample after every 1 hour dried it with filter paper and weighed (W_f_), evaluate water absorption by using Eq. (2)^4^.

2 $$\mathrm{Water \,\,Absorption }(\mathrm{\%}) =\frac{{\mathrm{w}}_{\mathrm{f}}-{\mathrm{w}}_{\mathrm{d}}}{{\mathrm{w}}_{\mathrm{d}}} \times 100.$$

Water retention was calculated by using Eq. (3). First weigh the dried lyophilized sample (W_D_) then immersed the sample (W_D_) in distilled water for 24 hours at 37 ℃ and weighed (W_S_) after every hour^5^.

3 $${\mathrm{Water\,\, retention }(\mathrm{\%}) =\mathrm{ w}}_{\mathrm{S}}-\frac{{\mathrm{w}}_{\mathrm{D}}}{{\mathrm{w}}_{\mathrm{S}}} \times 100.$$

Drug concentration was determined from calibration curve and % drug loading was calculated by using Eq. (4). Briefly triturated 10 mg sample was dissolved in 10 ml of acetylated methanol (5% acetic acid) solution centrifuged, supernatant solution was collected diluted with methanol and absorbance was measured at 305 nm^6^.

4 $$\mathrm{\% Drug\,Loading }= \frac{\mathrm{Calculated\,\, Drug \,\,content}}{\mathrm{Total \,\,amount \,\,of \,\,composite }} \times 100.$$

#### Structural characterization of CHG/Ag/nystatin composite

The chemical interaction was evaluated by Infrared spectroscopy (Alpha Bruker Platinum ATR, Germany). The surface morphology of both blank and loaded spongy composite was determined by scanning electron microscopy (SEM; JOEL; Japan). Briefly, the Sample was absorbed in liquid nitrogen, cut into cubes (3 × 3 × 1 mm) then observed at 15.0 kV^5^. The thermal stability of the composite was evaluated by differential scanning calorimetry (DSC) using Thermo-gravimetric analysis instruments software Universal analysis (version 4.5, USA model Q600 series) and Thermo-gravimetric analysis (TGA) using TGA module of thermal analysis instrument Q5000 series; Thermal analysis system (West Sussex, UK). X-ray powder diffraction analysis was done by using XRD; model JDX 3532; Japan.

#### Bioassays

##### Anti-microbial action

The bactericidal properties of polymeric composite having varying concentrations of Chitosan were evaluated by the zone of inhibition (ZOI) method. 107–108 CFU/ml suspension of S. aureus was proliferated on an agar plate. Samples were placed on plates & incubated for 24 hours at 37 ℃ then the zone of inhibition was measured^5^.

##### Anti-fungal action

Fungicidal properties of composite against fungal strains (*Candida* *Albicans*) were determined by the inhibition zone method.100 µl suspension of *C*. *Albicans* was spread on an agar plate. Four cups (6 mm) were bored using a cork borer and 100 µl of the sample was poured and plates were incubated for 24 hours at 25 ℃; then the zone of inhibition was measured^7^.

##### Cell viability studies

Cell viability of formulation was determined by using mouse skin (L929) fibroblast cells (Panjwani Centre for molecular medicine and Drug Research Karachi, Pakistan). Cells were seeded in a 96-well plate (3 × 105/well) in Dulbecco’s modified Eagle’s medium (100 μL) containing 0.01 g of sample. Medium was replaced with 10 µl MTT and 100 µl PBS solution after 48 hours then after 4 hours dimethyl sulfoxide and Formazan crystals were added and measure the absorbance at 490nm^5^.

#### In-vitro studies

Permeation investigation was done using “Franz Diffusion Cell” (TWJR-B) with 1cm^2^ diffusional area. Rat’s skin was placed in such a manner that the dermal side experiences receptor and the stratum corneum experiences the donor compartment. Place the 7.4 pH PBS solution in the receptor compartment and samples having an equivalent dose of 40 mg of nystatin in the donor compartment. Temperature and stirring were maintained at 32 ℃ and 700 rpm respectively. Draw the samples after different time intervals of up to 24 hours and replace them with an equal volume of phosphate buffer^8^. The drug release mechanism of different formulations (F1-F8) was evaluated using DD Solver Software.

#### Animal studies

To investigate results of CHG/Ag/Nystatin polymeric spongy composite wounds (burn) were created on white male albino rats (*Rattus norvegicus*) of age 90 to 100 days and weighed 200 - 250 gm after approval from the faculty of Pharmacy, Bahauddin Zakariya University, ethical committee Ref No 219/PEC/2022. All experimental research work was carried out following (Animal Research: Reporting of In-vivo Experiments) The ARRIVE Essential 10.

A total of 24 rats were allocated into four groups with six rats in each group, all rodents included in the study were healthy having a weight range of 200–250 g and age of 90–100 days kept in the natural cycle of day and light having free access to commercial food and water ad libitum one week before the study any animal injured or underweight and age excluded from the study. The second-degree burn was created by placing a hot stainless-steel rod (120 ℃) on the rat’s abdomen skin after anesthetized by sodium thiopental injection (50 mg/kg intra-peritoneally). An electric clipper was used to shave the dorsum and disinfect the area with an alcohol (70%) solution^9^.

Topical Treatment was applied in the following manners two times a day for 21 days of therapy. Drug administration started right after the burning procedure.

Group 1; control (untreated), Group II; Nystatin ointment. Group III; CHG/Ag; Group IV; CHG/Ag/Nystatin

Wound size was determined by measuring size through digital Vernier calliper everyday till the end of the study and percent wound closure was determined by using Eq. (5)^10^.

5 $$\mathrm{Wound \,\,Closure }(\mathrm{\%}) = 2 - \frac{\mathrm{ wound\,\, size}}{\mathrm{Initial \,\,Wound \,\,Size}} \times 100.$$

The rats were euthanized and skin excision (2 × 2 cm^2^ with the depth of full thickness) around the wound was made from two animals from each group on day 14 and rest on day 21. Skin samples were placed in Formalin buffer 10 % then embedded in paraffin, skin were marked with Hematoxylin-eosin and afterward with Masson Trichrome stain and degree of re-epithelization of wound was observed under light microscope^11^. All methods were carried out in accordance with relevant guidelines and regulations.

### Statistical analysis

The *in-vitro* studies were statistically analyzed by using analysis of Variance (ANOVA) test and Regression analysis. The comparison of antibacterial and antifungal activities of formulations was assessed with two way ANOVA followed by post-hoc Tukey’s test. All statistical assessment was accomplished by applying Graph Pad Prism using significance level at P > 0.05.

### Ethical statement

All animal studies were conducted after approval from departmental ethical committee Ref No 219/PEC/2022 and in accordance with the ARRIVE guidelines.

### Declaration

This article is derived from MPhil research project of Miss Asmara Ashraf D/O Muhammad Ashraf under registration number 2012-BZBP-104 submitted to Higher Education Commission repository.

## Results

**Figure 1:**
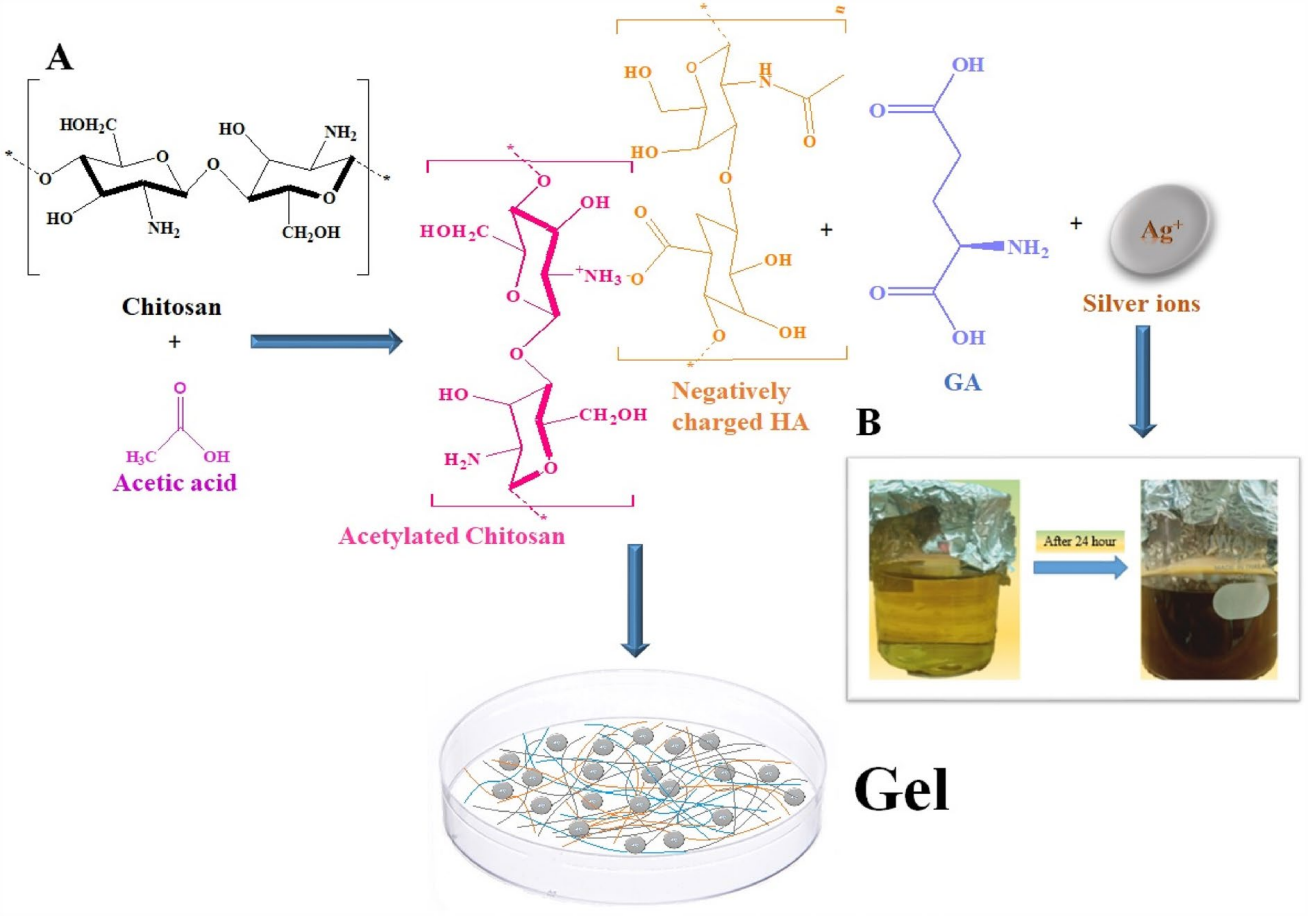
(**A**) Schematic representation of CHG/Ag/Nystatin composite, (**B**) change in color indicating formation of AgNps (*HA hyaluronic acid, *GA* glutamic acid).

In the present study, different formulations were fabricated using glutamic acid as a cross-linker, nystatin, and AgNps for the synergistic effect of formulations. An amidation reaction between l-Glutamic acid (Carboxyl group) and acetylated Chitosan (amino group) was produced. Hyaluronic Acid (H) binds to the –NH group of Glutamic Acid (Fig. 1). Color changes from light to pale yellow after the incorporation of AgNps and nystatin. The pH of all formulations ranges from 5.31 to 5.74, percent swelling was 37.50–108.75 % (Fig. S1), water retention was 6.97–23.07 % (Fig. S2) and porosity ranges from 33.80 to 67.50% (Table 1), Percent drug loading was 95–100% and rheological studies (Fig. S3) showed that all formulations had pseudo plastic behavior and followed non-Newtonian flow. The hydrophilicity of formulations increased as Chitosan concentration increased. This could be attributed to the formation of a loose polymeric network owing free space while decreased Chitosan concentration resulted in compact structure causing decreased ability for absorption and retention. This behavior explained previously^12^.

**Table 1:** pH, water retention, swelling ratio, and porosity studies of different formulations.

Trials	Chitosan (%)	Hyaluronic acid (%)	pH	% water retention	% swelling ratio	% porosity	% drug release
Fl	1.5	0.2	5.44 ± 0.07	23.07 ± 0.10	108.75 ± 0.56	67.5 ± 0.45	70.84 ± 0.06
F2	1.0	0.2	5.3 ± 0.03	26.60 ± 0.34	87.50 ± 0.57	59.6 ± 0.87	90.59 ± 0.07
F3	1.0	0.3	5.33 ± 0.01	18.36 ± 0.24	81.25 ± 0.37	50.6 ± 0.28	71.69 ± 0.09
F4	1.0	0.4	5.69 ± 0.08	13.04 ± 0.12	68.75 ± 0.67	48.4 ± 0.56	64.51 ± 0.02
F5	1.0	0.5	5.74 ± 0.05	12.04 ± 0.45	62.50 ± 0.26	42.0 ± 0.34	81.04 ± 0.05
F6	0.5	0.2	5.48 ± 0.04	10.11 ± 0.31	48.75 ± 0.56	37.8 ± 0.12	64.78 ± 0.07
F7	0.5	0.3	5.52 ± 0.06	8.04 ± 0.13	41.25 ± 0.64	36.2 ± 0.25	86.49 ± 0.09
F8	0.5	0.4	5.63 ± 0.04	6.97 ± 0.25	37.5 ± 0.59	33.8 ± 0.42	71.97 ± 0.04

**Figure 2:**
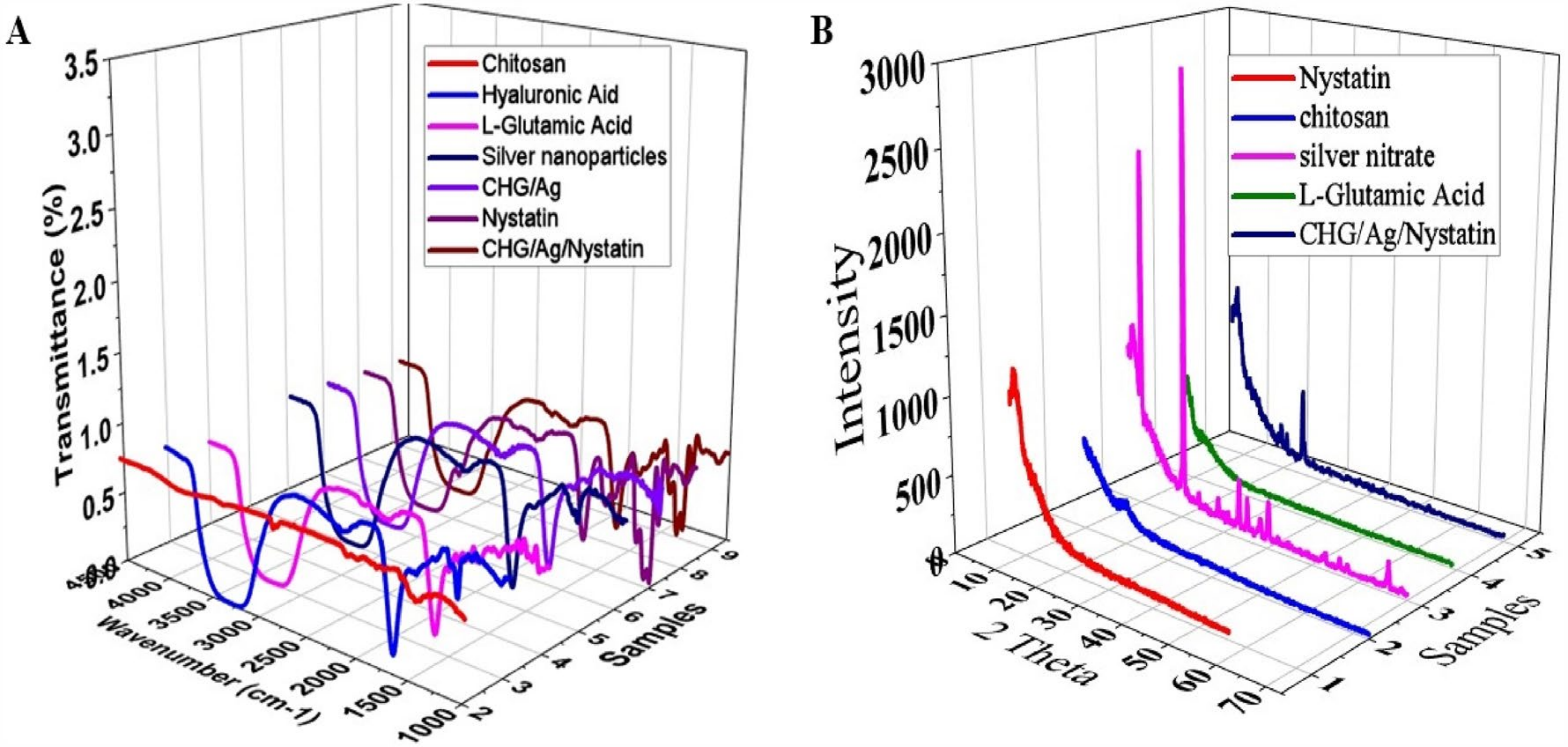
FTIR spectra of Chitosan, Glutamic Acid, Hyaluronic Acid, AgNps, CHG/Ag, Nystatin, and CHG/Ag/Nystatin (**A**); XRD comparative graph of silver nitrate, Chitosan, l-Glutamic Acid, Nystatin, CHG/Ag/Nystatin and Nystatin (**B**).

IR spectra of Chitosan and Hyaluronic Acid (Fig. 2A) showed peaks at 3350 cm^–1^, 2878.93 cm^–1^, and 1648.63 cm^–1^, 1553.84 cm^–1^, 1372.49 cm^–1^ and 1044.8 cm^–1^ due to stretching of –OH and N–H group; CH_3_ group; Carboxyl group stretching of amide I & II, flexing vibration of –OH group and C–O–C bond respectively, as reported in previous literature^13,14^. l-Glutamic Acid showed characteristic peaks at 2736.50 cm^–1^, 3335.44 cm^–1^, 1504.35 cm^–1^, and 1635.67 cm^–1^ due to C–H stretching; O–H stretching, stretching of N–H and C=O bond of the amino group respectively^15^. A weak band of the O-H group appeared at 1446.65 cm^–1^ due to the amino group of Chitosan and Carboxyl group of l-Glutamic Acid. Band at 1407.56 cm^–1^ appeared due to NH vibrations of Hyaluronic Acid in CHG composite^5,15^. AgNps showed peaks at 3303.42 cm^–1^,1636 cm^–1^, and 634.71 cm^–1^ due to N–H and O–H vibrations; stretching vibration of C=C and –C–C–H bonding respectively all these peaks of AgNps were in good accordance with previous literature^16^. Nystatin showed peaks at 3282.8 cm^–1^ ,1644.49 cm^–1^ ,1426.05 cm^–1^, and 1042.74 cm^–1^ due to –NH and –OH stretching; carboxyl ion (COO–); deformation of CH_3_ and Stretching vibration of CH, respectively^17^. Peaks at 1426.05 cm^–1^, 1644.49 cm^–1^ and 1048.92 cm^–1^ confirm the loading of nystatin in CHG/Ag/Nystatin composite without any interaction.

**Figure 3:**
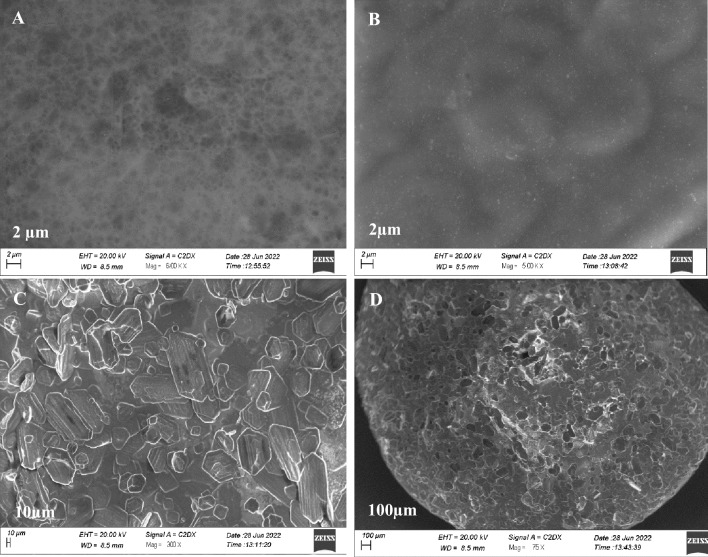
Morphological Structure of (**A**) (CHG composite), (**B**) (AgNps), (**C**) (CHG/Ag composite), (**D**) (CHG/Ag/Nystatin).

Morphological studies indicate the sponge-like structure and no phase separation of CHG composite (Fig. 3A). Synthesized AgNps are of uniform size (2 µm), and spherical in shape (Fig. 3B). Brightness in the structure showed the presence of AgNps, size of the composite increased from 10 to 20 µm after attachment AgNps (Fig. 3C) and further increase in size up to 100 µm after loading of nystatin (Fig. 3D). This study showed that interconnected porous structure promote cell attachment, remove pus cells thus encourage wound healing by providing suitable environment

**Figure 4:**
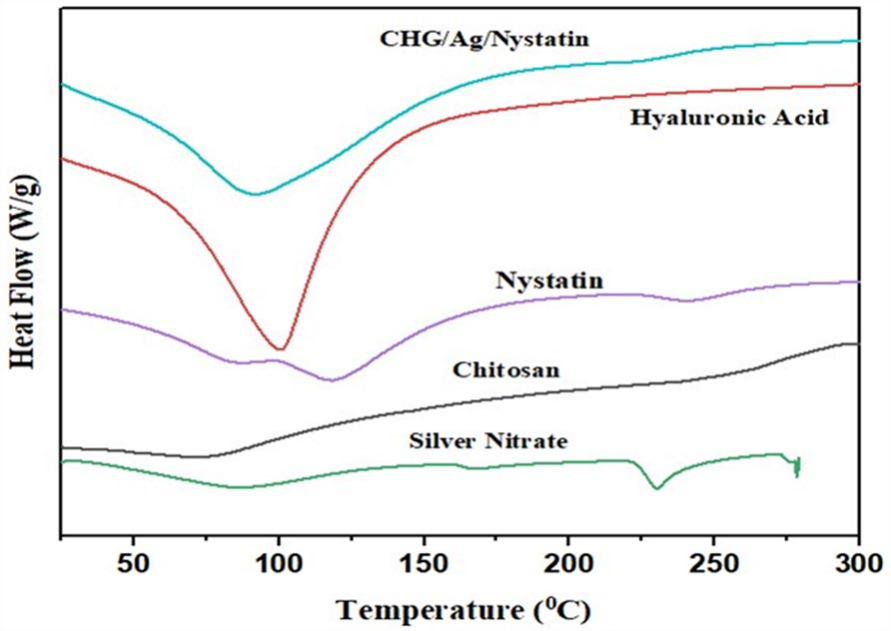
DSC (silver nitrate, chitosan, nystatin, hyaluronic acid, and CHG/Ag/nystatin).

Thermogravimetric analysis (Fig. 4) exhibits an endothermic peak of Chitosan showing the presence of moisture at 70.49 ℃ and polymer degradation peak at 245.48 ℃^18^. 15% weight loss occurs from 25 to 100 ℃ due to water evaporation, 8% weight loss was observed from 100 to 216 ℃ and about 28.52 % weight loss observed till 291 ℃^19^. Hyaluronic Acid showed an endothermic peak of moisture at 101.79 ℃ and an exothermic peak of polysaccharide degradation at 240 ℃^20^. Major weight loss was observed from 220 to 280 ℃ as a result of degradation^21^. An endothermic peak at 36.66 ℃ showed moisture in l-Glutamic Acid and melting at 205 ℃^22^. 1st weight loss observed at 194 ℃ and 85% weight loss till 327 ℃. CHG/Ag spongy composite showed a single endothermic peak at 132.94 ℃ and weight loss occur in three stages. 30 % and 20 % weight loss occur from 22 to 100 ℃ and 113.34 ℃ respectively and degradation occurs at 212.08 ℃. Nystatin showed an endothermic moisture peak at 115.13 ℃ and an endothermic peak at 242.87 ℃ due to melting^23^. For Nystatin 43 % weight loss was observed from 23 to 122 ℃ then 18% weight loss at 222–253.51 ℃. CHG/Ag/Nystatin showed a single endothermic peak at 92.83 ℃ because of evaporation. 48% weight loss due to moisture was observed till 100 ℃ then till 200 ℃ 15% weight loss was observed further constant weight shows the stability of the formulation. XRD analysis showed Hyaluronic Acid and Chitosan presented peaks at 10° and 20° with reflection planes of (110) and (220) indicating the anhydrous and hydrated crystals and 35-55° peaks show amorphous region. L-Glutamic Acid showed peaks at 10.3°, 20.0°, and 20.4° with reflection planes of (002), (004), and (110). Silver nitrate exhibited numerous strong peaks. Peaks at 38.14° and 44.20° assigned to the diffraction of crystalline Ag. AgNps showed peaks at 32.01°, 38.13°, 46.09° and 57.67° with the index plane of (101), (11), (200) and (220). CHG/Ag composite exhibits an amorphous structure with some peaks of crystalline Ag at 38.14° and 44.20°. Nystatin presented minor peaks at 13.86°, 20.48° and 20.00°^19,24–28^. CHG/Ag/Nystatin exhibited a stable amorphous structure of a spongy composite with some peaks of silver Nanoparticles. XRD analysis (Fig. 2B).

**Figure 5:**
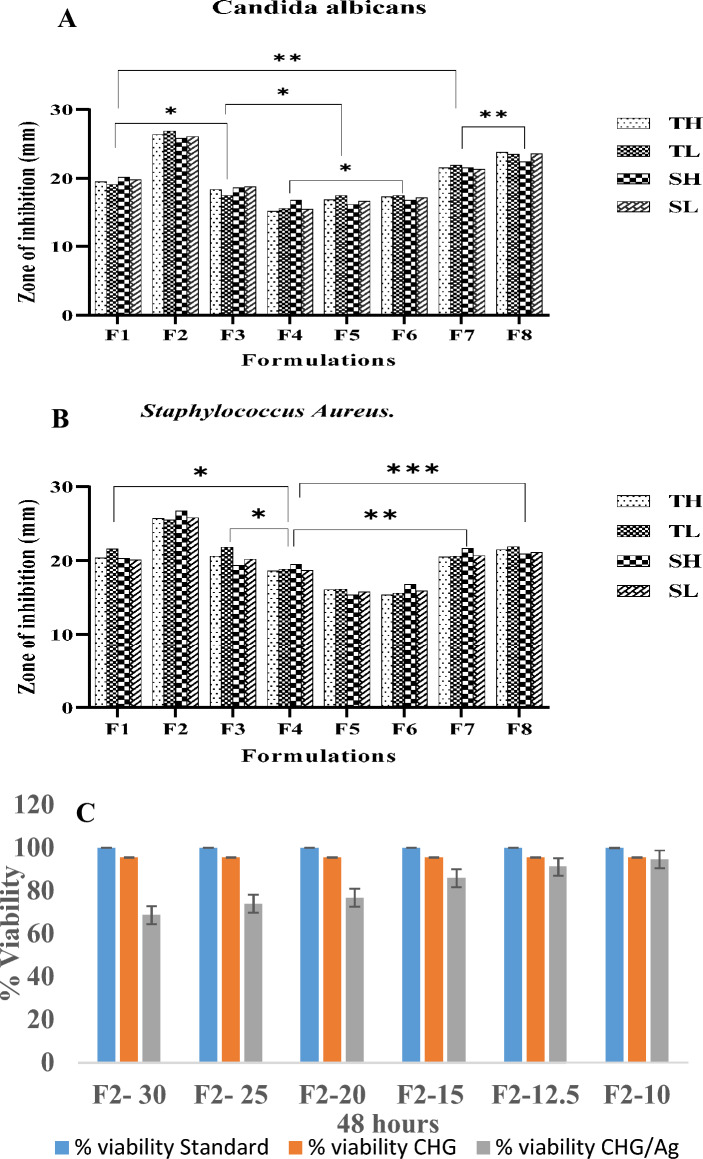
Significant and non- significant relation between formulations against Candida Albicans (**A**) Staphylococcus Aureus (**B**). *(*TH* tested high concentration, *TL* tested low concentration, *SH* standard high concentration, *SL* standard low concentration). Cytotoxic analysis of standard, CHG and CHG/Ag composite (**C**).

Cell viability of all formulations was in the range of 70–95 %. Formulation (F2) was declared as the safest among all with 95% viability having an AgNps concentration of 10 mg. It was found that an increase in the concentration of AgNps decreases cell viability from 94 to 68% (Fig 5C). This behavior could be due to apoptosis and alteration in the penetrability of the mitochondrial membrane^29^, while Hyaluronic Acid and Chitosan are considered economical, non-toxic and biodegradable polymers with > 80% cell viability^30^.

In-vitro drug release data (Fig. S4 & Table S1) showed that F2, F5, and F7 formulations had increased permeation as compared to F1, F3, F4, F6, and F8 formulations. This could be attributed to changes in Chitosan and Hyaluronic Acid concentration. F2 formulation was declared as an optimized formulation with 90% drug release within 12 hours following the Higuchi drug release mechanism. In the Korsmeyer Peppas model exponent (n) swelling mechanism was in the range of 0.14–0.25 which shows that the diffusion mechanism is Fickian, and no statistically significant difference was found as the p-value for all formulations was > 0.5. In-vitro data showed formulation having Hyaluronic Acid 0.02% & Chitosan 1% (F2) showed highest skin permeation. Due to the hydrophilic nature of Chitosan, absorb water, as Chitosan concentration increases swelling and porosity of formulations increase causing the drug released immediately. Likewise, the hydrophobic nature of Hyaluronic Acid causes decreased swelling capacity as the Hyaluronic Acid concentration increases. At low Chitosan and high Hyaluronic Acid concentration drug release is challenging and a very less amount of drug is released due to its compact structure. At high Chitosan and low Hyaluronic Acid concentration, drug release is maximum in a short period due to outbursts, and the therapeutic effect of the drug is lost. This manner of Hyaluronic Acid and Chitosan reported previously^31^.

Anti-microbial studies revealed that the zone of inhibition against Staphylococcus Aureus of formulations (F1–F8) ranges from 15.4 to 25.7 mm and for Candida Albicans 15.1 to 26.9 mm. F2 formulation showed best results 25.7 ± 0.06 for Staphylococcus Aureus and 26.9 ± 0.01 for Candida Albicans with significant p < 0.001 (Table S3 and S4) as compared to other formulations (Fig. 5A,B). This may be due to synergistic effect of Chitosan, Nystatin and AgNps. Previous researchers also investigated the bactericidal activity of Chitosan and AgNps. Anti-microbial effect of Chitosan was due to stable positive charge on nitrogen atom^32^. AgNps are non-toxic to human cells but destroy the micro-organisms by rupturing the outer membrane at low concentration^33^.

**Figure 6:**
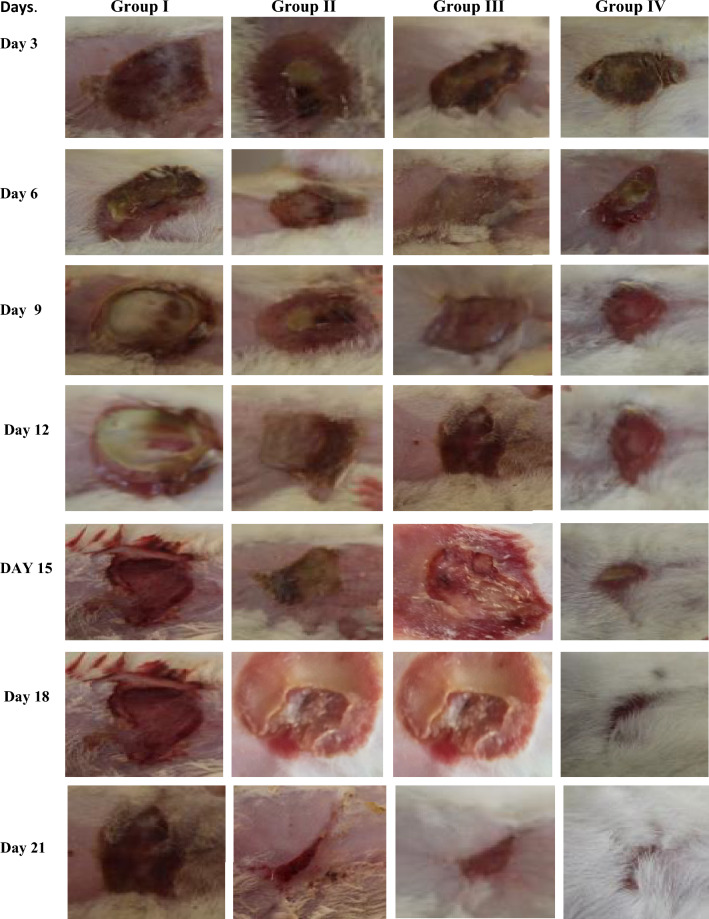
Images showing the wound size after every three days of different treatment groups.

Burn wound healing and change in wound size in different groups at different days given in Fig. 6 and Fig. S5 respectively. Results revelaed that F2 formulation cure wound in a statistically significant from 2.0 cm to 0.06 cm with in 18 days as compated to other grooups p < 0.001 while in CHG/Ag spongy composite and nystatin ointment group wound size decreased to 0.366 cm and 0.533 cm respectively after 21 days. These results suggested that CHG/Ag/Nystatin cure the wound in a more efficient manner due to synergistic effect of AgNps, nystatin and polymers. Wound healing was delayed in CHG/Ag group animals due to fungal infections and in nystatin ointment group wound did not heal properly due to bacterial infection. Wound healing ability of Hyaluronic Acid and Chitosan base carriers were also reported previously^34,35^.

**Figure 7:**
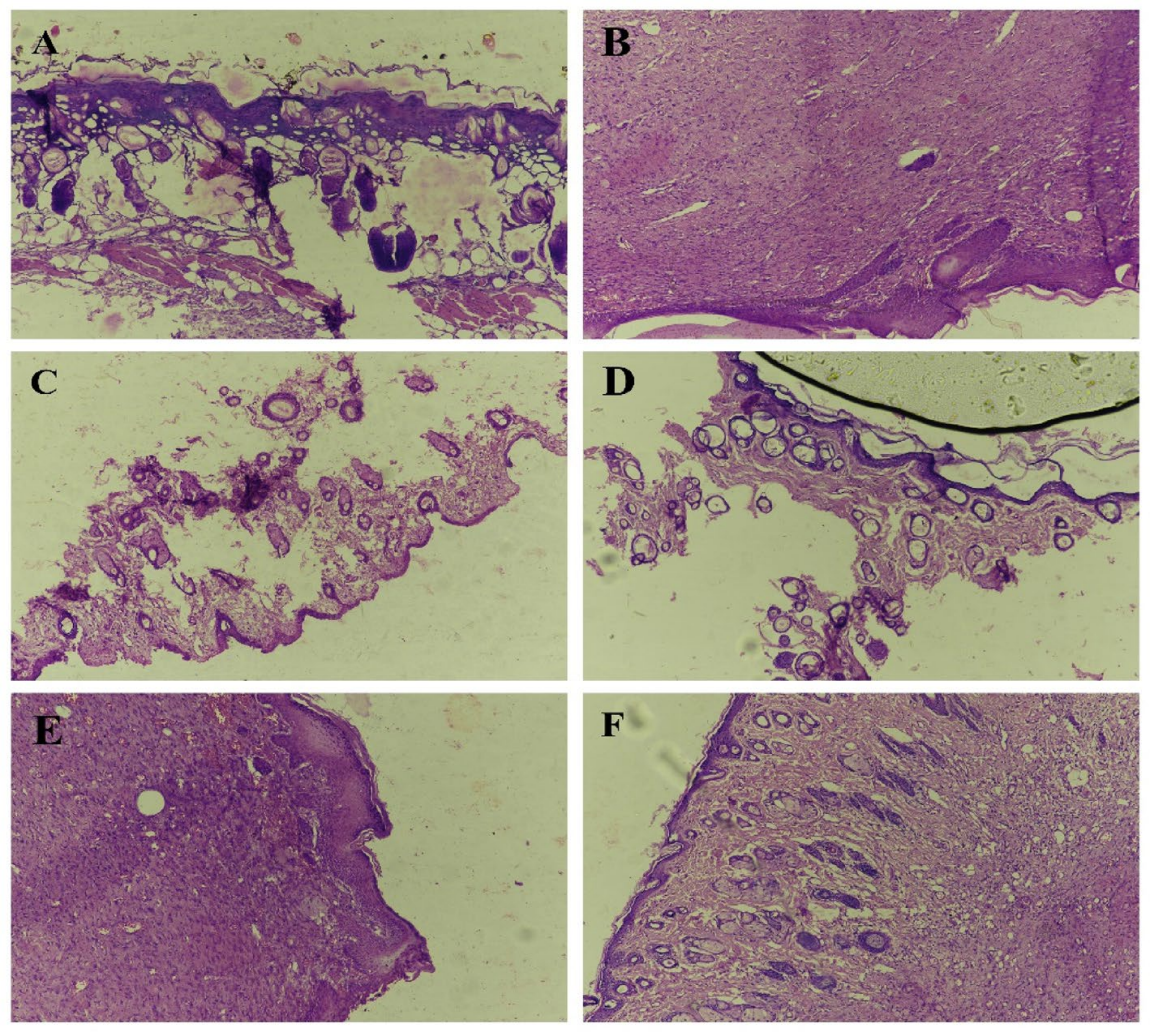
Histopathology of Skin after 14 and 21 days (**A**,**B**) CHG/Ag/Nystatin ointment; (**C**,**D**) Nystatin ointment; (**E**,**F**) CHG/Ag composite).

Histopathological studies showed that on day 14 animals received CHG/Ag/Nystatin ointment (Fig. 7A) there was an increase in the thickness of the epidermis with thick collagen fibers, and fibroblast was present with inflammatory response. The stratum spinosum was prominent and the stratum corneum was present without any detachment. The group received nystatin ointment (Fig. 7C) there was denaturation of collagen bundle fiber, and no prominent hemorrhagic response was observed. In CHG/Ag group (Fig. 7E) severe hemorrhagic response was observed beneath the basal layer, presence of a thick collagen bundle with distorted glandular structures on day 21. In CHG/Ag/nystatin ointment group (Fig. 7B) dermal and epidermal layers were intact with no separation, and collagen fiber was widely distributed along with the presence of normal sweat glands and hair follicles. In Nystatin ointment group (Fig. 7D) there was denaturation of collagen bundle fiber with no prominent hemorrhagic response was observed in CHG/Ag group (Fig. 7F) thick collagen bundles were intact, no prominent hemorrhagic and inflammatory response was observed, and hair follicles and sweat glands were normal group

Histopathological studies on second degree burn rat model showed that CHG/Ag/Nystatin composite had more effect on granulation and epithelization in comparison with other formulations and healed wound within 18 days. Formerly it was reported that Chitosan and Hyaluronic Acid had effect on wound healing. Loading of nystatin and AgNps in CHG composite causes Synergistic effect and promotes healing as compared to Nystatin ointment and CHG/Ag composite.

### Comparative analysis

The current study deals with the formation of polymeric composite with the loading of the drug Nystatin Till now, no research is reported for the loading of nystatin in CHG/Ag composite to enhance wound healing effect. As these polymers are cheap, biodegradable, and non-toxic, enhance the drug release, solubility, and stability of the drug and enhance wound healing as compared to other formulations. A study reported enhanced anti-microbial effect of drugs using Chitosan and Hyaluronic Acid network^36,37^.

### Inference

In a recent study novel polymeric spongy composite was fabricated loaded with AgNPs & Nystatin to improve wound healing in warm climatic conditions. Studies showed that Polymers and drugs inhibit bacterial and fungal infections and boost up the skin regeneration process. Burn wound studies on rats proved that optimized polymeric composite has improved and best wound healing as compared to other available commercial products.

### Supplementary Information

Supplementary Information.

## Data Availability

The datasets used and/or analyzed during the current study available from the corresponding author on reasonable request.
