# Supplementary material for: Synthesis and characterization of Hyaluronic Acid (HA) modified polymeric composite for effective treatment of wound healing by transdermal drug delivery system (TDDS)
Source: Sci Rep. 2023 Aug 17;13:13425. doi: 10.1038/s41598-023-40593-9 (PMC10435553; doi:10.1038/s41598-023-40593-9)

**Supportive data.**

Figure S 1: Swelling ratio of formulation (F1-F8).

Figure S 2: Water retention of formulations (F1-F8).

Figure S 3: Graph representing the viscosity of formulations (F1-F8) vs shear rate.

.


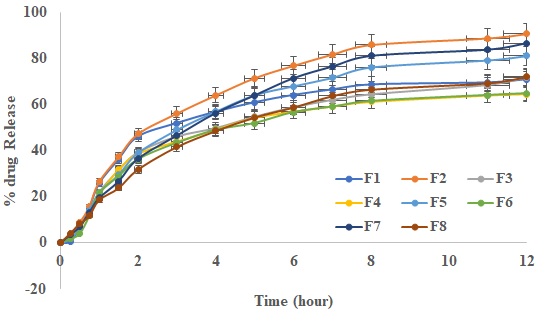


Figure S 4: % drug release of formulations (F1-F8).

Table S1: In-vitro drug release studies of CHG/Ag/Nystatin spongy composite (mean ±SD).


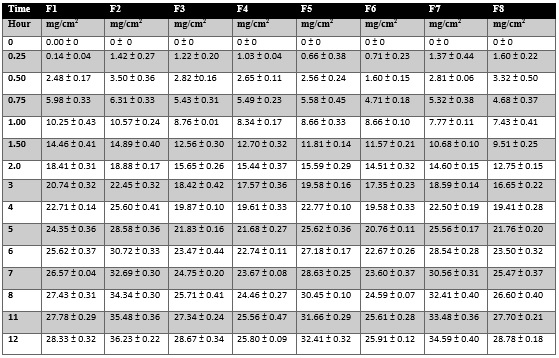


*
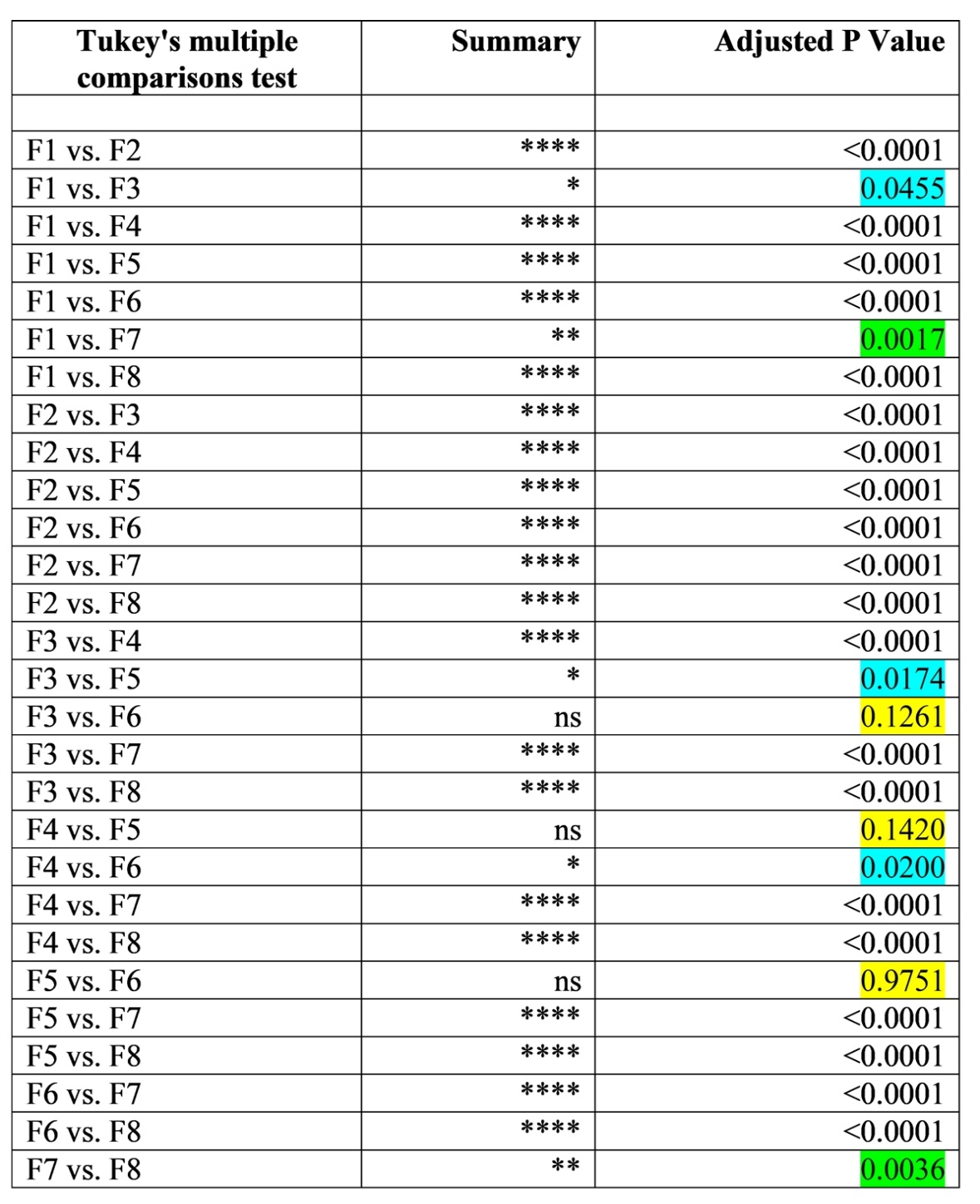
*Table S2: Formulation comparison test for candida Albicans.

Table S3: Formulation comparison test for Staphylococcus Aureus.
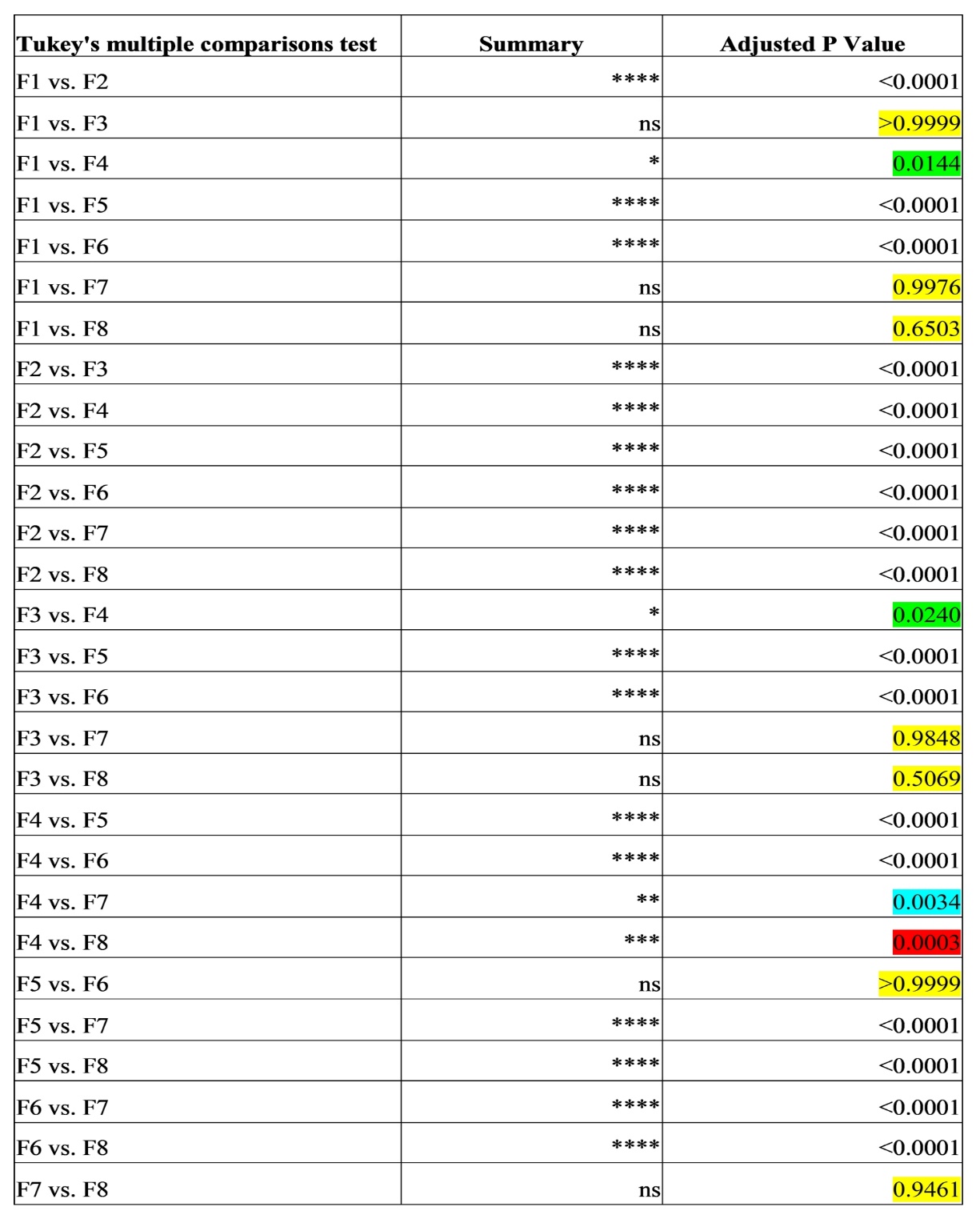


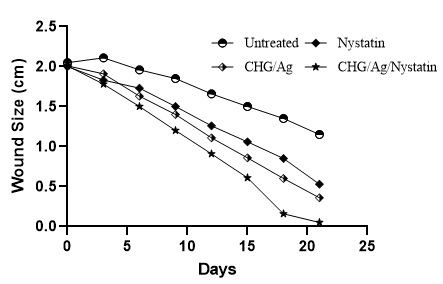


Figure S 5: Graph representing wound size after every three days of different treatment groups.

Table S4: %age Wound Closure of different treatment groups on different days.


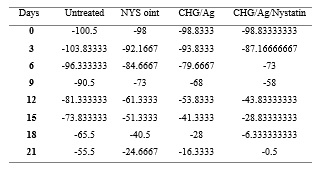

Supplement: Supplementary file 1 — Supplementary Information. [file 41598_2023_40593_MOESM1_ESM.docx]
